# T cell immunomodulation by clinically used allogeneic human cancellous bone fragments: a potential novel immunotherapy tool

**DOI:** 10.1038/s41598-018-31979-1

**Published:** 2018-09-10

**Authors:** Yasser M. El-Sherbiny, Jehan J. El-Jawhari, Timothy A. Moseley, Dennis McGonagle, Elena Jones

**Affiliations:** 1 National Institute of Health Research Leeds Biomedical Research Centre, Leeds Teaching Hospitals NHS Trust, Leeds, UK; 2 Clinical Pathology Department, Faculty of Medicine, Mansoura University, Mansoura, Egypt; 3 Leeds Institute of Rheumatic and Musculoskeletal Medicine, University of Leeds, Leeds, UK; 4 NuVasive, Inc., Biologics R&D, San Diego, CA USA

## Abstract

Multipotential stromal cells (MSCs) demonstrate strong immunomodulation capabilities following culture expansion. We have previously demonstrated that human cancellous bone fragments (CBFs) clinically used as viable allografts for spinal fusion have resident MSCs that exhibit T cell immunomodulation after monolayer expansion. This study investigated the immunomodulatory ability of these CBFs without MSC culture-expansion. CD4 positive T cells were induced to proliferate using CD3/CD28 stimulation and added to CBFs at different ratios of T cells per gram of CBF. A dose-dependent suppressive effect on T cell proliferation was evident and correlated with increased culture supernatant levels of TGF-ß1, but not PGE2. CBF-driven immunosuppression was reduced in co-cultures with TGF-ß neutralising antibodies and was higher in cell contact compared to non-contact cultures. CBF gene expression profile identified vascular cell adhesion molecule-1, bone marrow stromal antigen 2/CD317 and other interferon signalling pathway members as potential immunomodulatory mediators. The CD317 molecule was detected on the surface of CBF-resident cells confirming the gene expression data. Taken together, these data demonstrate that human clinically used CBFs are inherently immunomodulatory and suggest that these viable allografts may be used to deliver therapeutic immunomodulation for immune-related diseases.

## Introduction

In the last decade, cellular therapy such as multipotential stromal cells (MSCs) has been used extensively for immunomodulation in the variety of clinical settings including graft-versus-host disease (GVHD), Crohn’s disease, rheumatoid arthritis, kidney transplantation, type II diabetes and multiple sclerosis with promising outcomes^1–3^. MSCs are imbued with remarkable *in vitro* and *in vivo* immunomodulatory properties although initially defined based on their clonogenicity, high proliferative capacity and potential for trilineage differentiation to the bone, cartilage and fat lineages^4,5^. MSC immunomodulatory abilities include a substantial inhibition of stimulated CD4 or CD8 T-cell proliferation, suppression of proliferation and antibody formation by B cells, and modulation of the expansion as well as promoting the differentiation of monocytes into M2 macrophages with immunosuppressive phenotype^6,7^. Although available, MSC-based therapies require extensive controlled good manufacturing practice (GMP)-grade culturing and remain highly variable in terms of MSC tissue source, *ex vivo* manipulation, cell doses and methods of delivery. Additionally, intravenously injected cultured MSCs are known to be trapped in lungs^8^ whereas locally-delivered cells are rapidly degraded after administration^9,10^ and thus have a short time window for their immunomodulatory action.

We have previously shown that human cancellous bone fragments (CBFs) clinically-used as cellular bone allografts to augment bone regeneration primarily for spine fusion, contain bone-resident MSCs capable (after monolayer expansion) of the suppression of stimulated CD4+ T-cell proliferation, in addition to their classical MSC tri-lineage differentiation abilities^11^. These CBFs are produced from cadaveric human cancellous bone using extensive immuno-depletion bone washing procedures and are histologically characterised by an almost complete removal of blood-lineage cells from the bone marrow cavity. We have previously shown that these CBFs were also enriched for MSC-lineage cells including bone-lining cells and bone-embedded osteocytes. Phenotypically, enzymatically extracted cells from these CBFs contained high proportions of CD45^−^CD271^+^ cells^11^, a recognised phenotype of native bone-resident MSCs^12–14^. Based on this, we hypothesised that these CBFs could have an innate immunomodulatory activity partially related to MSC content. In support of this hypothesis, immunosuppressive effects of allogeneic bone grafts have been previously reported in several independent animal studies^15–17^. The aim of this study was, therefore, to examine the immunomodulatory capacity of these CBFs without any manipulation or MSC expansion, in co-cultures with allogeneic CD3/CD28-stimulated CD4 T cells.

We found dose-dependent suppression of CD4 T-cell proliferation and an increase in TGF-ß1 levels in these co-cultures, indicating an intrinsic immunomodulatory potential of CBFs. Gene expression analysis of CBFs prior to co-cultures provided a list of candidate immunomodulatory molecules potentially eliciting immunomodulation, with CD317 being confirmed at the protein level. Altogether, these findings suggest that these CBFs may potentially be used to elicit therapeutic immunomodulation in the clinical settings.

## Results and Discussion

### The effect of cancellous bone fragments (CBFs) on CD3/CD28-stimulated T-cell proliferation

**Figure 1:**
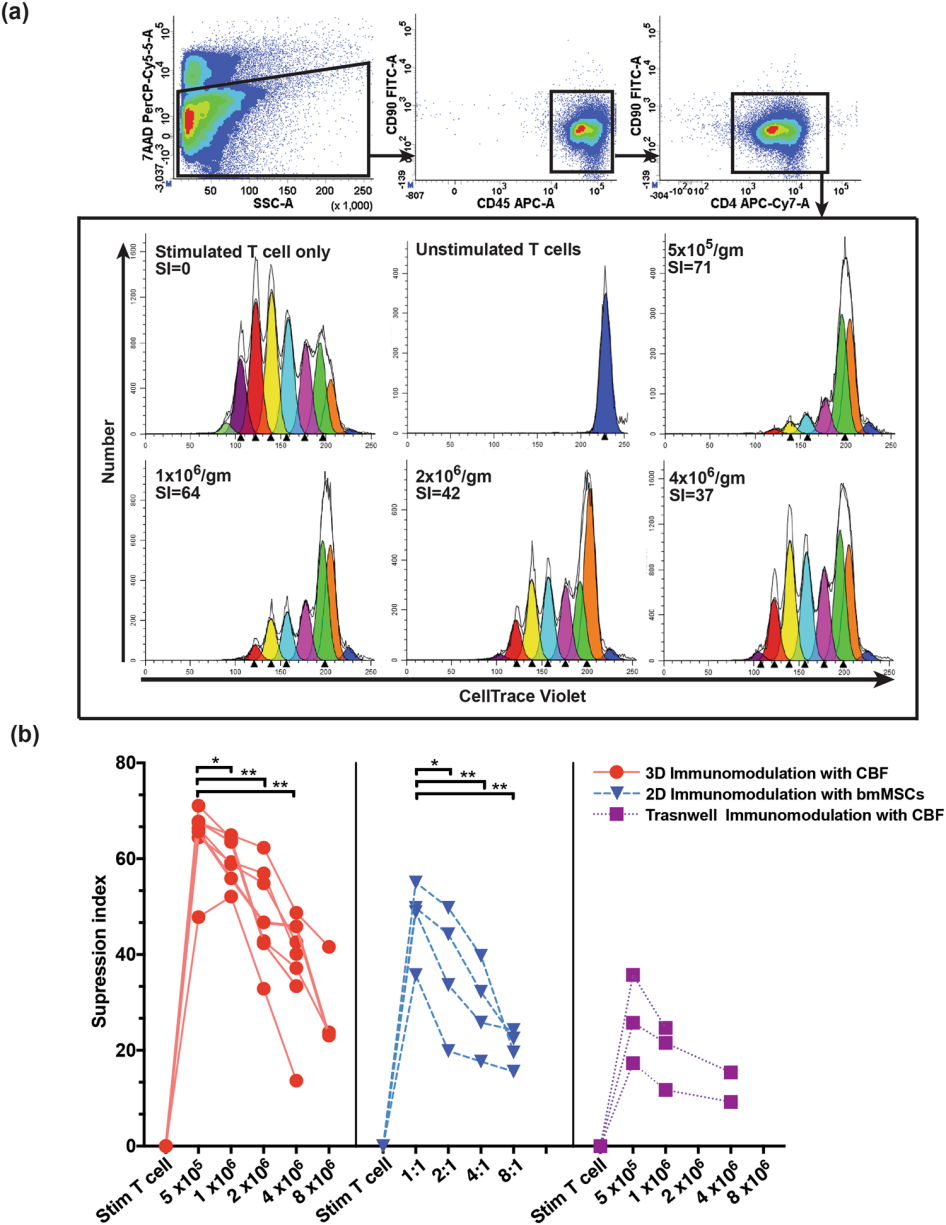
FACS gating strategy and experimental design of experiments to investigate the immunosuppressive expects of CBFs or bmMSCs on stimulated CD4^+^ T cells. (**a**) T cells present in the co-culture media were stained with antibodies against CD90, CD45 and CD4, and a viability dye 7AAD. Following the 7AAD/Side scatter gating for dead cell exclusion, CD45^+^CD90^−^CD4^+^ T cells were identified and analysed for proliferation suppression. Histograms representing the proliferation suppression analysis of CD45^+^CD90^−^CD4^+^ T cells cocultured for 5 days with 1 gm of CBFs (representative sample) at increasing T cell ratios. Colours represent cell generations detection, and calculated suppression index (SI) measured as % of suppression compared to no suppression control is indicated in the top left corner. (**b**) Comparison of T cell proliferation SIs in different co-culture conditions: 3D with CBFs (left panel), 2D with cultured bmMSCs (middle panel) and in transwell cultures with CBFs (right panel). Stim T cell – Stimulated T cell only (no suppression) control (Wilcoxon signed rank test, *p =  < 0.05, **p =  < 0.01).

The co-culture of MSCs with alloantigen- or CD3/CD28-stimulated T cells particularly purified CD4 T cells is a standard assay to study immunomodulatory effects of MSCs on the adaptive immune cells^11,18–20^. In these assays, T cell:MSC ratios are set at 1:1 to 10:1 T cells per MSC. The same assay was applied in our CBF experiments, but the co-cultures were set up based on the addition of different numbers of activated CD4 T cells to tissue culture wells containing pre-weighted CBFs of the same weight. The reason for this method is that potential immunomodulation by CBFs could not be solely attributed to MSCs present in CBFs and the other cells such as osteoprogenitors, osteocytes and the residual immune-lineage cells were present. On day 6 of co-culture, CD4 T cells were collected from culture supernatants and their proliferation suppression was analysed by flow cytometry following gating on the CD45^+^CD90^−^CD4^+^ population (Fig. 1a, top panel). Proliferation suppression was measured as T cell suppression index (SI), based on the degree of cell tracer dye dilution. Control culture wells included unstimulated T cells and stimulated T cells, both without CBFs. As expected, control stimulated T cells displayed multiple divisions (no suppression); this was assigned a T cell suppression index of zero (Fig. 1a, lower panel). As expected, no dye dilution was evident in unstimulated T cells control (Fig. 1a, lower panel). The highest suppression (median SI of 71%) was observed at the lowest T cell:CBF ratio (5 × 10^5^ T cells/gm CBF) (Fig. 1a, lower panel). With the increase of T cell: CBF ratios, a corresponding decrease in the average T-cell suppression index was detected (Fig. 1a, lower panel), which reached medians of 71, 64, 42 and 37% for 0.5, 1, 2, and 4 × 10^6^ T cells/gram of CBFs respectively (Fig. 1a, lower panel). Statistical analysis showed significant differences in suppression indices between different T cell: CBF ratios (Fig. 1b, left panel). This indicated the ability of CBFs to suppress the proliferation of CD4 T cells in a dose-dependent manner.

To illustrate the magnitude of CBF modulation on CD4 T cell proliferation in relation to standard 2D cultured MSCs, bone marrow-derived MSCs (bmMSCs) were used instead of CBFs and co-cultured with purified stimulated CD4 T cells at 1:1, 2:1, 4:1, and 8:1 ratios^11^. The average 2D bmMSC-mediated T cell suppression indices had medians of 49, 39, 29 and 21% for T cell:MSC cell ratios of 1:1, 2:1, 4:1 and 8:1, respectively, and significantly different between the ratios (Fig. 1b, middle panel). These results demonstrated that in our experimental conditions, 1 gm of CBFs provided CD4 T cell immunosuppressive effects corresponding to approximately 1.5 to 2 × 10^6^ of 2D cultured bmMSCs.

### Soluble factors involved in CBF immunomodulation

To test if the suppression of CD4 T-cell proliferation can be induced via CBF secreted factors without their contact with T cells, the co-cultures were performed without direct contact between the T cells and CBFs. For this, CBFs and T cells were separated by transwell cell culture inserts. The results showed that although the T cell suppression indices were higher in T cell: CBF co-cultures compared to stimulated T cell positive control (zero SI), T cell suppression indices were on average 2.8-fold lower compared to contact co-cultures (Fig. 1b, right panel). This indicated that soluble factors contributed to the observed immunomodulation, but they were not exclusive and cell contact-mediated mechanisms of immunosuppression were also involved.

**Figure 2:**
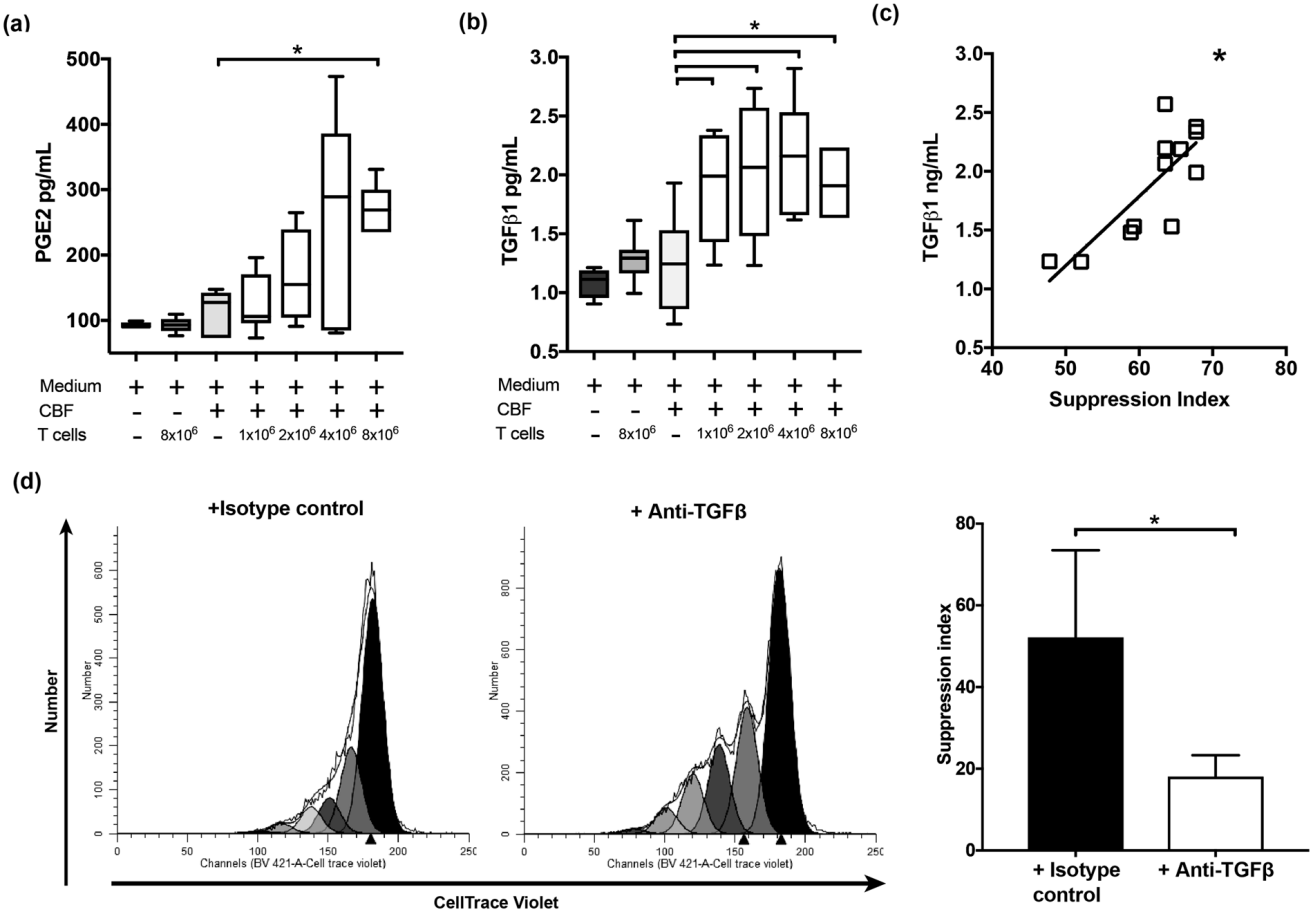
PGE2 (**a**) and TGF-ß1 (**b**) levels in the supernatants of co-cultures of CBFs and T cells. A stepwise increase in TGF-ß1 concentrations with increased numbers of T cells compared to CBF only (Mann-Whitney *p =  < 0.05) and other controls, in contrast to PGE2 where these trends were less obvious. (n ≥ 7) in all co-culture ratios and (n = 3) in 8 × 10^6^ T cells/gm of CBF. (**c**) Positive correlation between TGF-ß1 and T-cell SI in co-cultures of T cells and CBFs (spearman rho r = 0.673, p = <0.05). (**d**) CBF-driven immunomodulation in the presence of TGF-ß neutralising antibodies or isotype control antibody (co-culture ratio: 1 × 10^6^ T cell/gm CBF) fluorescence histograms for a representative sample (left) and combined data (n = 3, right, paired t test, *p = <0.05).

PGE2 and TGF-ß1 are two well recognised soluble mediators of immunomodulation by MSCs on T cells^1,6,21^. To explore their involvement in CBF mediated T cell immunomodulation, co-cultures were set similarly so that the increasing numbers of T cells were added to the same weights of CBFs. Control wells included: media only, CBF in media only, and stimulated T cells only. Although PGE2 levels in these co-cultures were above those of media only and T cells only controls (Fig. 2a), they were in the lower range of PGE2 concentrations previously described for 2D cultured MSCs^22,23^ and showed no significant correlation with T cell proliferation suppression indices from the same cultures.

In contrast, significantly higher levels of TGF-ß1 in the supernatants from co-cultures of CBF with T cells at different ratios compared to all controls including CBF only controls were observed (Fig. 2b). TGF-ß1 levels were comparable to previously published for 2D MSC cultures^22,24,25^ and positively correlated with CBF T cell immunomodulation (r = 0.673, p = 0.019) (Fig. 2c).

To confirm the mediating role of TGF-ß1, the effect of CBFs on CD3/CD28 stimulated T-cell proliferation was examined in the presence of TGF-ß neutralising antibody or isotype control and we used ratio 1 × 10^6^ T cell/gm CBF for this experiment. The cell tracer peaks indicated more proliferation of T cells in the presence of TGF-ß neutralising antibodies compared to CBFs with isotype control antibody (Fig. 2d, left panel) and the suppression indices in co-cultures of CBFs and stimulated T-cells in the presence of TGF-ß neutralising antibodies were markedly lower compared to isotype control antibody (p = <0.05, Fig. 2d, right panel).

### Immunomodulatory gene expression profile of the CBFs

As our co-culture data showed a possibility for both cell contact-mediated and soluble factor-mediated immunosuppression by CBFs, we next performed gene expression analysis in CBFs, in order to identify potential molecular candidates. Compared to fibroblasts, a common comparator for MSCs^26^, the top 50 highly-expressed genes in CBFs included mesenchymal lineage transcripts previously shown to be expressed in uncultured CD45^−^CD271^+^ bmMSCs and reflective of their multipotential nature (approximately 20% transcripts, Table 1). Interestingly, a similar number of genes (10 genes or 20%) were categorised as immune response-associated molecules, two of which (Vascular cell adhesion molecule-1 (VCAM1) and S100 calcium binding protein A8 (S100A8)) were previously shown to be expressed in CD45^−^CD271^+^ MSCs^27–29^. VCAM1 is considered an essential adhesion molecule mediating MSC:T cell contacts during immunosuppression^30^. The roles of defensins and alarmins (S100 proteins), innate immune defence molecules also highly expressed in CBFs (Table 1), remain poorly understood. However, recent evidence suggests that they may be mediating the homing and maintenance of hematopoietic progenitors in the vicinity of bone marrow stroma^27,31^. Several variants of osteopontin were highly expressed in CBFs; apart from being a well-known bone structural protein, osteopontin is also known as a regulator of the cross-talk between innate and adaptive immunity affecting T cell polarisation^32^. Interestingly, up-regulation of the osteopontin expression was previously noted in 3D cultured MSCs compared to their 2D counterparts^33^. Finally, CBFs also expressed higher levels of prostaglandin-endoperoxide synthase 2 (also known as Cox-2), an enzyme involved in the production of PGE2 that, in addition to immunosuppression, has a role in the osteoblast maturation^34^.

**Table 1:** MSC-specific and immune related molecules from the list of 50 top highly expressed genes in CBFs compared to fibroblasts.

Gene name	Gene code	Fold-Difference	Reference
***Native MSC*** **-** ***specific molecules***			
Secreted phosphoprotein 1 (osteopontin), transcript variant 2	*SPP1*	119.7	^28, 41^
Secreted phosphoprotein 1 (osteopontin), transcript variant 1	*SPP1*	64.1	^28, 41^
Insulin-like growth factor binding protein 3, transcript variant 1	*IGFBP3*	38.1	^28, 29, 41^
Bone gamma-carboxyglutamate (gla) protein (osteocalcin)	*BGLAP*	36.7	^28, 41^
Lipoprotein lipase (LPL)	*LPL*	33.9	^28, 29, 41^
Mesenteric estrogen-dependent adipogenesis	*MEDAG*	30.6	
SPARC-like 1	*SPARCL1*	26.1	^28^
Fatty acid binding protein 4, adipocyte	*FABP4*	25.8	^28, 41^
Insulin-like growth factor binding protein 3, transcript variant 1	*IGFBP3*	25.4	^28, 29, 41^
***Immune related molecules***			
S100 calcium binding protein A8 (S100A8)	*S100A8*	62.8	^28^
Interleukin 6 (interferon, beta 2)	*IL6*	61.8	
Prostaglandin-endoperoxide synthase 2 (prostaglandin G/H synthase and cyclooxygenase)	*PTGS2*	40.7	
Defensin, alpha 1	*DEFA1*	40.3	
Vascular cell adhesion molecule 1	*VCAM1*	25.8	^27– 30^
Cathelicidin antimicrobial peptide	*CAMP*	24.3	
Interferon stimulated exonuclease gene 20 kDa	*ISG20*	24.2	
Defensin, alpha 1	*LOC653600*	23	
Defensin, alpha 3, neutrophil-specific	*DEFA3*	22.5	
S100 calcium binding protein A12	*S100A12*	22.4	

Immune related molecules were classified based on gene ontology and KEGG pathway analysis using STRING. Native MSC-specific molecules were selected based on published literature.

**Figure 3:**
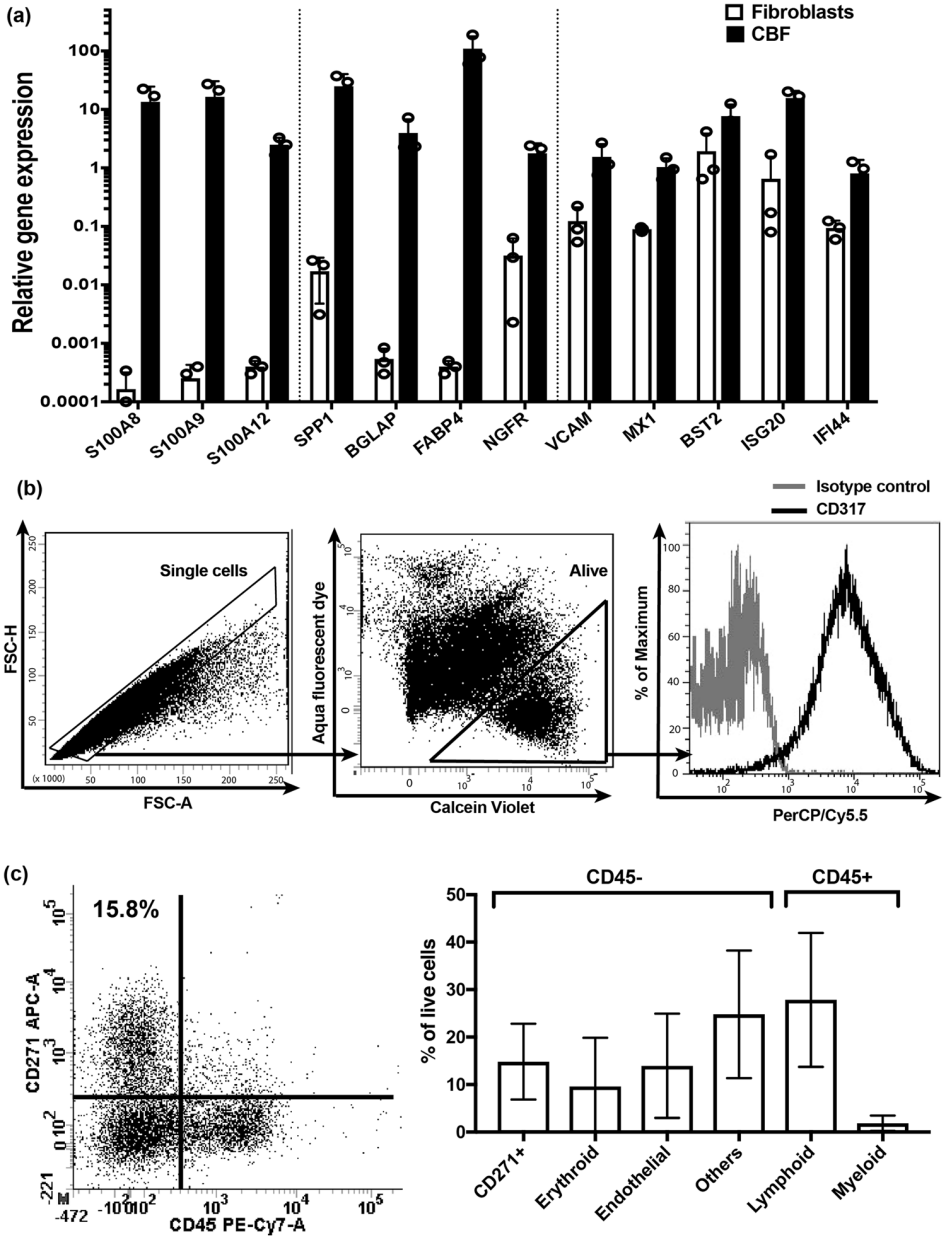
The analysis of candidate immunomodulatory molecules and cell populations in CBFs prior to immunomodulation experiments. (**a**) Immune-related and native MSC-specific gene expression by qPCR (bars represent mean values for n = 3 samples of CBFs and fibroblasts. (**b**) Gating strategy for flow cytometry detection of cell surface CD317 expression on enzymatically-extracted CBF-resident cells. Overlaid histograms for isotype control (grey line) and CD317 antibody (black line) on live single cells. (**c**) Cell populations released from CBFs and identifying CD45^−^CD271^+^ native MSCs and other lineage cells present in CBFs. FACS plot showing a representative sample (left) and combined data (n = 4, right).

To validate the gene array data, we tested the expression levels of selected genes using real-time PCR (Fig. 3a). The expression levels of all molecules selected for validation including native MSC specific molecules: *SPP1*, *BGLAP*, *FABP4*, and immune-related molecules: *S100A8*, *VCAM1*, *ISG20* and *SA10012*, were considerably higher in CBFs compared to fibroblasts. The higher expression of *NGFR*, a gene encoding CD271 protein was used as a positive control and indicated the presence of native MSCs in CBFs (Fig. 3a). Additionally, S100A9 was tested as a marker of myeloid-derived suppressor cells^35^, another candidate immunomodulatory cell potentially present in CBFs, and showed high upregulation in CBFs compared to fibroblasts (Fig. 3a).

To get additional insights into CBF-specific immune-related molecules, all immune-related transcripts with at least 2-fold higher expression in CBFs compared to fibroblasts were further scrutinized for functional gene annotation clustering using the Database for Annotation, Visualization and Integrated Discovery (DAVID)^36^. The transcripts falling into an immunity cluster were next divided into ‘secreted’ and ‘other’ groups (Table 2). Notably, several molecules belonging to the interferon signalling pathway previously described as potentially mediating immunomodulation^37^ and bone turnover^38^ were found in the ‘others’ group (Table 2). Interestingly, *MX1* gene belonging to the interferon signalling pathway family has been previously described as a specific marker of osteolineage restricted MSCs in mice^39^, and found to be nearly 10-fold higher in CBFs compared to fibroblasts (Fig. 3a). The expression of bone marrow stromal antigen 2 (BST2)/CD317, in particular, was higher in CBFs compared to fibroblasts by both microarray and qPCR (Table 2 and Fig. 3a). This molecule has been previously shown to be expressed on bone marrow stromal cells^40^ and more recently, on their particular immunomodulatory subset^37^. To investigate the presence of CD317 on the surface of CBF-resident cells, we performed flow cytometry on cells enzymatically released from CBFs using collagenase. Following sequential gating on single alive cells, we confirmed positive BST2/CD317 protein expression on cell surface of three CBF samples tested (Fig. 3b).

**Table 2:** All immune-related transcripts with at least 2-fold higher expression in CBFs compared to fibroblasts derived from Gene ontology analysis using DAVID.

Gene name	*Gene code*	Fold-Change
***Immune response: secreted***		
S100 calcium binding protein A8	*S100A8*	62.8
Interleukin 6	*IL6*	61.8
Defensin alpha 1	*DEFA1*	40.3
Complement factor D	*CFD*	31.6
Cathelicidin antimicrobial peptide	*CAMP*	24.3
Defensin alpha 3	*DEFA3*	22.5
S100 calcium binding protein A12	*S100A12*	22.4
Lipocalin 2	*LCN2*	14
Peptidoglycan recognition protein 1	*PGLYRP1*	12
Complement factor B	*CFB*	6.8
Ficolin 1	*FCN1*	6.8
CD14 molecule	*CD14*	5.1
Defensin alpha 4	*DEFA4*	4.9
Haptoglobin	*HP*	4.8
High mobility group box 1	*HMGB1*	4.5
Lysozyme	*LYZ*	4.4
Complement factor H	*CFH*	4.3
C-C motif chemokine ligand 5	*CCL5*	4.2
Ribonuclease A family member 4	*RNASE4*	3.6
Interleukin 1 receptor type 1	*IL1R1*	3.6
Complement C1q B chain	*C1QB*	3.5
Lipopolysaccharide binding protein	*LBP*	3.3
Bactericidal/permeability-increasing protein	*BPI*	3.2
Interleukin 18 binding protein	*IL18BP*	2.9
Ribonuclease A family member 3	*RNASE3*	2.4
***Immune response: others***		
Interferon stimulated exonuclease gene 20 kDa	*ISG20*	24.2
Myxovirus resistance 1, interferon-inducible protein p78	*MX1*	8.7
2′-5′-oligoadenylate synthetase 2, 69/71 kDa (OAS2)	*OAS2*	8.54
CD55 molecule, decay accelerating factor for complement	*CD55*	6.7
B-cell CLL/lymphoma 6 (zinc finger protein 51)	*BCL6*	4.9
Complement component 1, s subcomponent (C1S)	*C1S*	4.9
Hect domain and RLD 5	*HERC5*	4.7
CD300 molecule-like family member g (CD300LG)	*CD300LG*	4.4
V-ets erythroblastosis virus E26 oncogene homolog 1	*ETS1*	4.2
Bone marrow stromal antigen 2, Tetherin (CD317)	*BST2*	3.3
Mitogen-activated protein kinase kinase kinase 8	*MAP3K8*	3.1
DEAD (Asp-Glu-Ala-Asp) box polypeptide 3	*DDX3*×	3
Interferon induced transmembrane protein 1	*IFITM1*	3
Interferon induced with helicase C domain 1	*IFIH1*	3
Sequestosome 1	*SQSTM1*	3
FYN oncogene related to SRC, FGR, YES (FYN)	*FYN*	2.8
Interferon regulatory factor 7	*IRF7*	2.8
TANK-binding kinase 1	*TBK1*	2.7
Adenosine deaminase, RNA-specific, B1	*ADARB1*	2.7
Interferon-induced protein with tetratricopeptide repeats 2	*IFIT2*	2.6
Interferon-induced protein with tetratricopeptide repeats 3	*IFIT3*	2.6
Radical S-adenosyl methionine domain containing 2	*RSAD2*	2.5
Tripartite motif-containing 56	*TRIM56*	2.5
Yamaguchi sarcoma viral related oncogene homolog	*LYN*	2.4
PR domain containing 1, with ZNF domain	*PRDM1*	2.4

Immune related molecules with over 22-fold higher levels compared to fibroblasts are also present in Table 1 (highly expressed).

We were mindful that CBFs contained residual cells of other lineages on the bone surface, which could be eliciting immunomodulation. Consistent with our previous findings^11^, CBFs used in immunomodulation experiments contained cells with the phenotype of native bmMSCs (CD45^−^CD271^+^), which represented approximately 16% of all live cells enzymatically extracted from these CBFs [median (90% CI) 15.8% (5.3, 53.5), n = 9] (Fig. 3c, left panel). These cells also expressed classical MSC markers CD73, CD90 and CD105 (medians of 98, 99 and 73% of CD45^−^CD271^+^ cells, respectively) confirming their MSC identity. Using the distinct phenotypes to identify different cell lineages, the relative proportions of endothelial-lineage cells (CD45^−^CD271^−^CD31^+^), erythroid-lineage cells (CD45^−^CD271^−^CD235a^+^), myeloid-lineage cells (CD45^+^CD271^−^CD33^+^), and lymphoid-lineage cells (CD45^+^CD271^−^CD3/19^+^) were next analysed (Fig. 3c, right panel). Lymphoid-lineage cells were found to be more abundant than other lineages while myeloid-lineage cells were least prevalent (Fig. 3c, right panel). Because of very low numbers of these myeloid-lineage cells and to maximise the use of sample, it was not feasible to include other markers or testing for exploring the presence of myeloid-derived suppressor cells (MDSCs) as reported^35^ in these CBFs. We also found a population of cells lacking the chosen markers (labelled as others) which could represent more mature MSC-lineage cells such as osteoblasts and adipocytes (Fig. 3c, right panel).

CD45^−^CD271^+^ MSCs express genes characteristic of both stromal fibroblasts and pericytes^14,41^. In addition to other unidentified CD45^−^ cells, CBFs contain osteocytes inside the bone itself^11^. Osteogenically-differentiated MSCs *in vitro* have been reported to be immunomodulatory^42,43^. however, this has yet to be investigated for non-cultured osteocytes. Isolation and gene expression study of pure osteocytes, osteo- and adipogenically committed cells, MSCs and residual cells including MDSCs in CBFs would, therefore, be needed to address the relative contributions of different cell types to CBF gene expression pattern, and CBF immunomodulation in general.

In conclusion, this study is the first demonstration that natural 3D structures containing native uncultured MSCs possess high potential for immunomodulation. As such, these clinically available cellular allografts can be considered as a potential novel immunotherapy tool. As stated, several animal model studies have previously reported the ability of allogenic bone to prevent the rejection of transplanted organs^15,17^. In this context, our study demonstrated that clinically used CBFs could provide an ‘immuno-privileged niche’ capable of controlling adaptive immune cell responses in the allogeneic settings. These CBFs could, therefore, be further explored as local modulators of various inflammatory and immune-mediated diseases particularly those dependent on the CD4 T cell response. Further studies are needed to address the relative contributions of the different cell populations in CBFs including MSCs, osteocytes and other stromal- and immune-lineage cells, and also the potential effects of matrix-embedded proteins.

There is already good evidence for the safety of CBF implants in clinical practice since over 250,000 of these allografts have been used successfully in bone fusion procedures without any reports of tissue rejection in the clinical literature summarised in^11^. We propose that these allografts could be repurposed to provide site-specific immunomodulation, for example by being locally implanted within a permeable barrier (mesh), which can allow free movement of cells and signals. Our previous study has shown the expansion of MSCs in CBFs in long-term *in vitro* culture^11^ suggesting that CBF-driven immunomodulation may be maintained longer than a standard short ‘window of opportunity’ common for transplanted 2D cultured MSCs. Future study is needed to investigate the functional longevity of CBF immunomodulation including a possibility of protracted release of soluble immunosuppressive factors such as TGF-β1 and osteopontin from the bone matrix^32,44^.

In addition to therapeutic implications, this study proposes a new, standardised 3D *in vitro* model to investigate the interface between bone surface-resident cells and the immune system^45^ For example, their roles in immune cell retention and transit^46,47^, skeletal ageing^48^ or bone marrow response to infections^49^. Given the highly-controlled method of CBF tissue processing, these questions can be systematically addressed *in vitro* and *in vivo*, and provide future insights into the physiology of bone marrow microenvironment in humans.

## Materials and Methods

### Ethics statement

Written informed consent was obtained from every study participant before the sample was taken and research was carried out in compliance with the Helsinki Declaration. The bone marrow samples used to extract MSCs for this study were collected under ethical approval, 06/Q1206/127, National Research Ethics Committee Yorkshire and Humber–Leeds East, and peripheral blood was collected under the study number 04/Q1206/107. All experiments were performed in accordance with relevant guidelines and regulations.

### Cancellous bone fragments (CBF) processing

Cancellous bone fragments (Osteocel® allograft cellular bone matrix, 5 cc) from 15 different research consented donors (Nuvasive, San Diego, CA, USA) were prepared, according to The American Association of Tissue Banks (AATB) standards, by the manufacturer using extensive washes in order to remove the bulk of blood-lineage cells and cryopreserved^11^. For determination of cellular composition, CBFs were digested using 0.25% collagenase (Stem Cell Technologies, Grenoble, France)^11^ and the released cells were stained with antibodies against CD271-APC (Miltenyi Biotec), CD90-FITC, CD105-PE (both from BioRad, UK), CD73-PerCP-Cy^TM^5.5 and CD45-PE-Cy7 (both from BD Biosciences) as well as aqua fluorescent dye and Calcein AM from the live/dead violet viability/vitality kit (Thermo Fisher Scientific-Invitrogen) in order to identify native bone-resident MSCs^11^. Following gating for single cells, these MSCs were identified as reported before^11,14,41^ and recorded as a proportion of total live cells. Other CD45-negative CBF-resident cells were identified as: erythroid-lineage (CD235a^+^) and endothelial-lineage (CD31^+^), whereas other hematopoietic CD45^+^ cells were identified and subdivided into lymphoid-lineage (CD3+ and CD19+) and myeloid-lineage (CD33+). The antibodies used were: CD3-FITC, CD19-FITC, CD271-PE-Vio®770, CD31-APC and CD235a-APC-Vio®770 (all from Miltenyi Biotec), CD33-Alexa Fluor®700 and CD45-BUV395 (both from BD Biosciences). BST2/tetherin expression on different lineage cells was measured using CD317-PerCP-Cy^TM^5.5 (from BioLegend, UK). The data acquisition was performed on LSRII 4 laser flow cytometer (BD Biosciences), and the analysis was conducted using FACS DIVA software (BD Biosciences).

### CD4 T cells isolation, labelling and stimulation

To ensure consistency between experiments utilising different donor-derived CBFs, peripheral blood samples were taken from a single consented healthy donor. We have selected purified CD4+ T cells based on ISCT recommendation for testing the potency of MSC-related immunomodulation^50^. CD4 T cells were isolated using the two-step CD3^+^CD4^+^ T cell isolation kit (Miltenyi Biotec, Surrey, UK) via the depletion of non-CD4 cells followed by depletion of CD25^+^ T_regs_. Purified T cells were labelled with the carbocyfluorescein succinimidyl ester dye analogue, CellTrace™ Violet (Thermo Fisher Scientific), and stimulated to proliferate using Anti-Biotin MACSiBead™ particles preloaded with biotinylated CD2, CD3, and CD28 antibodies (Miltenyi Biotec); these were used at bead:T cell ratio of 2:1 for best stimulation condition in the presence or absence of CBFs, as described below.

### Co-cultures of CBFs and T cells

The CBFs (replicates of the same weight of approximately 250 mg) were placed in 24-well standard tissue culture plates and co-cultured with raising numbers of T cells. The ratios were corresponding to 0.5, 1, 2, 4, and 8 × 10^6^ T cells/gram of CBF in a total volume of 1 mL of medium per well. The medium was a 1:1 mixture of Glutamax™ RPMI 1640 medium (Thermo Fisher Scientific) supplemented with 10% FCS (Sigma-Aldrich, Dorset, UK) and StemMACS NH medium (Miltenyi Biotec), and the co-cultures were maintained in this medium for 5 days. For T cell proliferation suppression experiments, control culture wells (without CBFs) included: unstimulated T cells and stimulated T cells. In some experiments, anti-TGFß neutralising antibody or IgG1 isotype control antibody (R&D Systems) was added to cultures (ratio 1 × 10^6^ T cell/gm CBF) at 5 μg/ml at the start of co-culture and kept for five days. For non-contact transwell experimental conditions, transparent PET membrane cell culture inserts were used, 1.0 μm pore size, 1.6 × 10^6^ pores/cm^2^ (Corning) to allow maximum diffusion and separation between the T cells and CBFs.

For the analysis of T cell proliferation suppression, culture media was separated from CBFs, and T cells were isolated from culture media by pipetting and centrifugation and stained with 7AAD, CD90-FITC, CD45-APC and CD4-APC-Vio770. Following sequential gating for CD45^+^CD90^−^ cells (non-MSCs) and then CD4^+^ T cells (Fig. 1a), the T cell proliferation was analysed using ModFit software version 3.2 (Verity Software House, Topsham, ME. USA) algorithm for cell generations boundaries detection and computing. T cell division index (DI) was calculated as the total number of T cell divisions divided by T cell number at the start of culture^51^. The T cell proliferation suppression index was adapted from some previous studies^52,53^ and calculated according to the following formula:

$$T\,cell\,proliferation\,suppression\,index=100\times (\frac{1-DI(CBFs+T\,cells)}{DI(T\,cell\,stimulated\,only)})$$

### Co-cultures of 2D culture-expanded MSCs and T cells

To assess the extent of CBF-driven immunomodulation in comparison with standard 2D-cultured MSCs, bmMSCs (n = 4, passage 4–6) from healthy donors were used instead of CBFs in co-cultures with purified stimulated CD4 T cells as described above. Before co-cultures, the purity of 2D-cultured MSCs was confirmed using CD73-PE, CD90-FITC, CD105-APC, CD45-PE-Cy7, CD14-PE, and HLA-DR-APC-H7. MSCs were co-cultured with T cells in at ratios 1:1, 2:1, 4:1, and 8:1 for 5 days as described before^11^ then T-cell suppression index was calculated to measure inhibition of T cell proliferation.

### Measuring PGE2 and TGF-ß1 in co-culture supernatants using ELISA

The supernatants of bone/T-cell co-cultures were collected at the end of five days and kept frozen (−80 °C). The levels of PGE2 and TGF-ß1 were measured using ELISA kits (R&D systems) according to the manufacturer’s recommendations and the optical readings were taken using MULTISCAN EX reader equipped with Ascent software (Thermo electron corporation). Control wells included media, CBF-conditioned media and stimulated T cells in the absence of CBFs.

### Assessment of CBF immunomodulatory gene expression profile

To investigate whether CBFs express immunomodulatory genes before their contact with T cells, total RNA from 6 CBF samples was extracted using RNeasy Mini Kit (Qiagen, Germantown, MD) and used to generate a gene expression profile for each sample using the Illumina Bead Chip (HumanHT −12 v3). Human skin fibroblasts RNA (Cell Applications, Inc., CA, USA) was used as a control. Data analysis included gene identifiers, normalization and log2 transformation for signal values for each gene and hypothesis testing using one-way ANOVA or t-Test as appropriate. The top 50 molecules significantly over-expressed in CBFs compared to fibroblasts were assigned into categories based on Gene ontology and KEGG pathway analysis using the STRING database^54,55^. Additionally, all transcripts significantly over-expressed in bone fragments (over 2-fold difference) were further examined for functional annotation clustering using DAVID and the molecules appearing in the Immunity cluster were further subdivided into ‘secreted’ and ‘other’ groups based on individual protein’s information provided by STRING.

To validate gene array data, the quantitative RT-PCR assays were performed for some transcripts comparing CFBs and fibroblasts. The RNA was extracted from the digested CFBs and cultured fibroblasts using Single Cell RNA purification kit (Geneflow Ltd, Lichfield, UK). The complementary DNA (cDNA) was produced using high capacity reverse transcription kit (Thermo Fisher Scientific). The TaqMan probes (Thermo Fisher Scientific) for were used for the genes: Secreted phosphoprotein 1 (*SPP1*) HS00959010_m1, Bone gamma-carboxyglutamate protein (*BGLAP*) HS01587814_g1, Fatty acid binding protein 4 (*FABP4*) HS00609791_m1, Tetherin (*BST2*) Hs00171632_m1, Interferon-stimulated gene 20 (ISG20) Hs00158122_m1, Interferon-induced GTP-binding protein (*MX1*) Hs00895608_m1, Vascular cells adhesion molecules (*VCAM1*) Hs01003372_m1, Nerve Growth Factor Receptor (*NGFR*) Hs00609976_m1, (*S100A8*) Hs00374264_g1, (*S100A9*) Hs00610058_m1 and (*S100A12*) Hs00942835_g1. The PCR assays were run in duplicates using 384-well plates on QuantStudio™ 7 Flex Real-Time PCR System (Thermo Fisher Scientific). The gene expression levels were assessed relative to the housekeeping gene, hypoxanthine guanine phosphoribosyl transferase (*HPRT1*) HS99999909_m1.

### Statistical analysis

The statistical analysis and figures’ preparation was performed using GraphPad prism 6 software. The comparative statistical tests were chosen according to the data normal distribution tested using the Shapiro-Wilk normality test and included Spearman rho for correlation, Mann Whitney tests, one-way ANOVA or t-Test were used as appropriate. Any difference between the groups was considered as statistically significant when the p value < 0.05.

## Data Availability

The microarray gene expression array data that support the findings of this study is available from NuVasive, Inc., but restrictions apply to the availability of this data, which was used under license for the current study, and so is not publicly available. The data is however available from the corresponding author upon reasonable request and with permission of NuVasive, Inc.
